# Outbreaks of Gastroenteritis Due to Norovirus in Schools and Summer Camps in Catalonia, 2017–2019

**DOI:** 10.1128/spectrum.00119-22

**Published:** 2022-05-11

**Authors:** Ignacio Parrón, Irene Barrabeig, Núria Soldevila, Rosa Bartolomé, Susana Guix, Cristina Rius, Thais Cornejo-Sánchez, Conchita Izquierdo, Àngela Domínguez, Working Group for the Study of Outbreaks of Acute Gastroenteritis in Catalonia

**Affiliations:** a Agència de Salut Publica de Catalunya, Barcelona, Spain; b Department of Medicina, Universitat de Barcelona, Barcelona, Spain; c CIBERESP, Instituto de Salud Carlos III, Madrid, Spain; d Hospital Universitari Vall d’Hebrón, Barcelona, Spain; e Agència de Salut Pública de Barcelona, Barcelona, Spain; f Universitat Pompeu Fabra, Barcelona, Spain; Johns Hopkins Hospital

**Keywords:** norovirus, outbreak, summer camps, schools, calicivirus infection, delayed reporting, size of the group, seasonality

## Abstract

We studied outbreaks of acute gastroenteritis due to norovirus in schools and summer camps during 2017–2019 in Catalonia (Spain). The overall attack rate was 21.27% in schools and 33.42% in summer camps (RR 0.64 [95% CI 0.58–0.70]) and 52.63% of outbreaks occurred in cold months and 47.37% in warm months. The mean delay in reporting was 5.61 days (SD 5.58 days) and the mean duration was 6.11 days (SD 6.08 days), with a Pearson correlation coefficient of 0.84 (*P* < 0.001) between these variables. In outbreaks with person-to-person transmission, the aOR was higher the longer the delay in reporting: 3.07 (95% CI 1.21–7.81) when the delay was 5–8 days and 3.81 when it was >9 days (95% CI 1.42–10.23). The cold months posed a higher risk than the warm months. In common source outbreaks the risk was higher in children in secondary-higher education and in summer camps.

**IMPORTANCE** Norovirus is the main cause of viral acute gastroenteritis outbreaks worldwide. The low infectious dose and the lack of long-term immunity in infected persons means that norovirus often causes outbreaks in institutions and closed and semiclosed centers. Norovirus gastroenteritis are usually mild, with no complications, but occasionally can result in hospital admission. Understanding the risk factors involved in a norovirus outbreak can reduce the spread, severity, and duration of the outbreak and, when a vaccine becomes available, this understanding would help us identify the population groups need to get vaccinated. Here, we show the outbreaks due to norovirus in schools and summer camps, the correlation between the delay in reporting and duration of outbreaks and the relationship of the attack rate and the size of the groups.

## INTRODUCTION

Acute gastroenteritis (AGE) is the second leading cause of death from infectious diseases in children aged < 5 years worldwide (1, 2). The agents most frequently involved are viruses, mainly of the *Caliciviridae* family. In 2011, human caliciviruses caused 71,000 deaths worldwide in children aged < 5 years (3). In 2019, the World Health Organization (WHO) estimated that norovirus was the most frequent cause of foodborne illness in the European Region with 15 million of cases (4). In the United States, norovirus is responsible for > 60% of cases of AGE (5) and is estimated to be the cause of 90% of all viral AGE outbreaks worldwide (6). AGE norovirus outbreaks often occur in closed or semiclosed institutions such as nursing homes, summer camps, nurseries, schools, and cruise ships, where transmission is easy, either person-to-person through close contact or from exposure to contaminated food, water, or surfaces (6, 7).

In the United States, 14 million children and adults attend summer camps annually (8). AGE norovirus outbreaks are frequently reported in US summer camp users. Between 2009 and 2016, 63% of AGE outbreaks in summer camps were due to norovirus (9). The activities and accommodation of summer camps, infrastructure deficits in the supply of drinking water and the lack of health education of users and staff may be facilitating factors (9).

Norovirus is also a common cause of outbreaks in schools. In England from 2014 to 2019, 18.4% of all suspected or confirmed norovirus outbreaks occurred in educational settings (10). In Shanghai in 2016 and 2017 there were 215 outbreaks of norovirus reported, 87.91% (189 outbreaks) of them occurred in schools and kindergartens (11). In the United States between 2013 and 2018 there were 1409 norovirus outbreaks in schools and universities affecting 83,669 people (12). In 2017, in Catalonia (Spain), school was the setting for 35% of outbreaks occurred in closed and semiclosed facilities (13).

Norovirus infections occur at any time of the year, although it is reported that the incidence of isolated cases and outbreaks is higher during the cold months (14). The low infective dose (mean of 18 viral particles) and the lack of long-term immunity in infected people mean that noroviruses frequently cause epidemic outbreaks (6). Water and food control, hand hygiene and surface disinfection are common preventive measures (15). However, the high resistance of the virus to chlorine (16), heat (17), organic solvents and acidic pH (7, 17) limit the effectiveness of these measures.

The objective was to analyze outbreaks of AGE due to norovirus that occurred in schools and summer camps in 2017–2019 in Catalonia and identify the factors associated with their appearance and extent.

## RESULTS

In the study period, 101 outbreaks in which norovirus was identified as the causative agent were reported: 37.6% (38 outbreaks) occurred in schools or summer camps, with 5,165 exposed people and 1,295 affected people (AR 25.07%). There were 12 outbreaks in 2017 with 2,120 exposed people and 598 affected people (AR 28.21%),11 outbreaks in 2018 with 1026 exposed people and 271 affected people (AR 26.41%) and 15 outbreaks in 2019 with 1,927 exposed people and 417 affected people (AR 21.64%). Thus, there was a downward trend in attack rates during the study period (Chi square of linear trend 22.82; *P* < 0.001).

Of the 38 outbreaks, 19 occurred in schools with 3,549 exposed people and 755 affected people (AR 21.27%) and the remaining 19 in summer camps with 1,616 exposed people and 540 affected people (AR 33.42%). The ratio of these rates (RR) was 0.64 (95%CI 0.58–0.70); *P* < 0.001. Person-to-person transmission occurred in 23 outbreaks (60.53%) and common source transmission in 15 (39.47%), of which transmission was due to well water in one outbreak and was foodborne in the rest.

**FIG 1:**
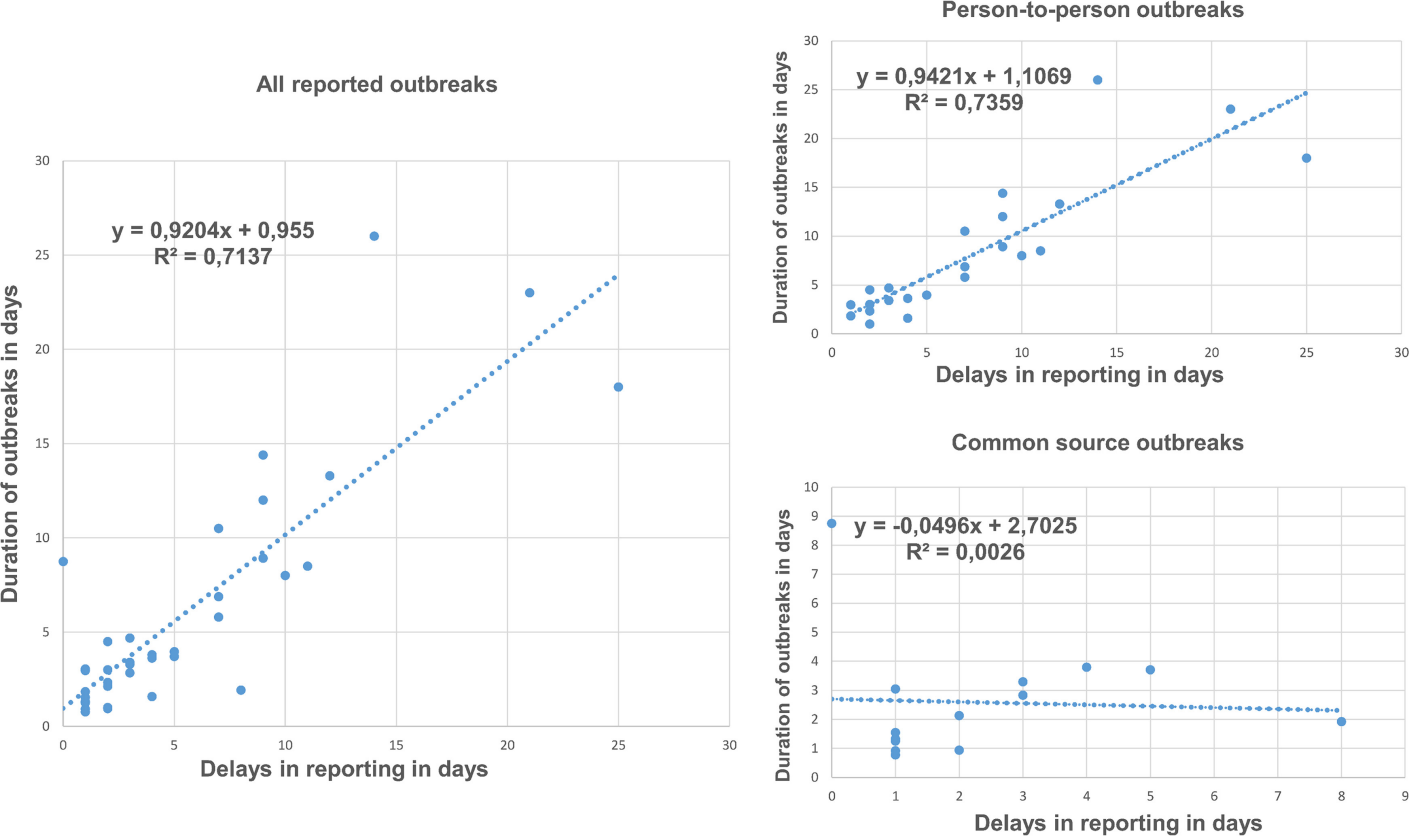
Correlation between delay in reporting and duration of outbreaks in total outbreaks and by type of transmission.

The AR was 19.19% (591/3079) in outbreaks of person-to-person transmission and 33.75% (704/2086) in outbreaks of common source transmission (RR 0.57 [95% CI 0.52–0.62]; *P* < 0.001). Twenty outbreaks occurred in the cold autumn or winter seasons (52.63%) and 18 in the warm spring or summer seasons (47.37%). The time from symptom onset in the first case to its reporting to the Epidemiological Surveillance unit ranged from 0 to 25 days (mean 5.61 days, SD 5.58 days). The duration of outbreaks from symptom onset of the first case to symptom onset of the last case ranged from 1 to 14 days (mean 6.11 days, SD 6.08 days). The delay in reporting and the duration of the outbreak showed a linear correlation (Pearson's correlation coefficient 0.84; 95% CI 0.72–0.92; *P* < 0. 001) for all outbreaks and for outbreaks with person-to-person transmission (Pearson's correlation coefficient 0.86 [95% CI 0.69–0.93]; *P* < 0.001), but not for common source transmission (Pearson's correlation coefficient −0.05 [95% CI −0.57 to 0.49]; *P* = 0.86). (Fig. 1).

**FIG 2:**
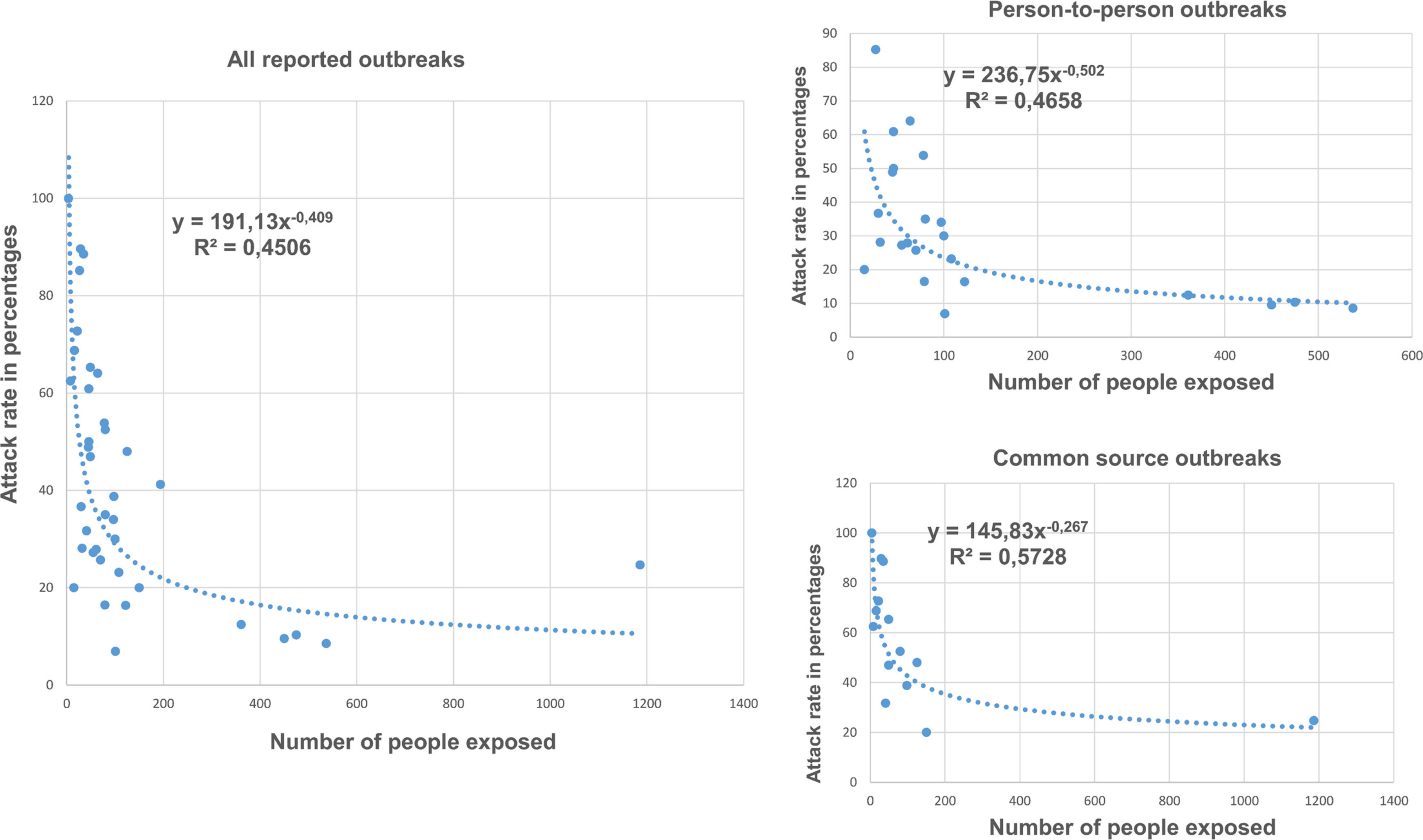
Correlation between number of exposures per outbreak and the attack rate observed in all outbreaks together and by type of transmission.

The mean number of exposures was 135.9 people per outbreak (SD 217.6) and the overall attack rate was 41.65%. The mean number of people affected per outbreak was 34.1 (SD 46.2), with the number of people affected ranging from 3 to 293. Considering all outbreaks together, a decreasing exponential relationship was observed between the number of people exposed and the attack rate (Fig. 2) and in the log-log model the Pearson correlation coefficient was −0.6713 (95% CI −0.8159 to −0.4477; *P* < 0.001) and −0.6825 (95% CI −0.8544 to −0.3761; *P* < 0.001) in outbreaks with person-to-person transmission and −0.7568 (95% CI −0.9186 to −0.3781; *P* = 0.002) in common source outbreaks.

We surveyed 636 exposed persons, of whom 420 were affected (66.04%). Table 1 shows the raw and adjusted ORs according to sex, educational level, type of center, season of the year, delay in reporting and type of transmission in the subjects surveyed. We detected an interaction between the type of transmission and the type of center and the season of the year, so common source and person-to-person transmission outbreaks were analyzed separately.

**TABLE 1:** Raw and adjusted odds ratios of the association between the variables considered and the risk of becoming ill in all outbreaks

Variable	Ill	Not ill	Total	OR^*a*^	95% CI	aOR^*b*^	95% CI
Sex							
Male	188	61	249	2.06	1.45–2.93	1.67	1.10–2.52
Female	232	155	387	1		1	
School level							
Infant (>2 and <7 yrs)	183	70	253	1		1	
Primary (≥7 and <13 yrs)	106	6	112	6.76	2.84–16.09	6.95	2.69–17.94
Secondary and higher (≥13 and <19 yrs)	63	17	80	1.42	0.78–2.59	1.51	0.71–3.22
Adults (≥19 yrs)	68	123	191	0.21	0.14–0.32	0.23	0.14–0.37
Center							
School	237	147	384	1		1	
Summer camp	183	69	252	1.18	1.06–1.31	3.55	1.96–6.44
Season							
Warm months	164	108	272	1		1	
Cold months	256	108	364	1.56	1.12–2.17	1.67	1.09–2.56
Reporting delay							
≤ 2 days	143	99	242	1		1	
>2 days and ≤4 days	74	44	118	1.16	0.74–1.83	3.08	1.60–5.92
>4 days and ≤9 days	119	46	165	1.79	1.17–2.74	5.25	2.88–9.58
>9 days	84	27	111	2.15	1.30–3.56	7.11	3.62-13.95
Type of transmission							
Common source	133	90	223	0.65	0.46–0.91		
Person-person	287	126	413	1			

*^a^*OR, raw odds ratios.

*^b^*aOR, adjusted odds ratios.

In outbreaks with person-to-person transmission (Table 2) the risk of becoming ill was associated with being a primary school child, the outbreak occurring in a summer camp during the cold months of the year, and delayed reporting. The association between delayed reporting and the risk of becoming ill showed a dose-response relationship. Taking as a reference outbreaks reported in ≥ 2 days, the aOR was 3.07 (95% CI 1.21–7.81) for 5 to 9 days and 3.81 (95% CI 1.42–10. 23) for > 9 days.

**TABLE 2:** Raw and adjusted odds ratios of the association between the different variables considered and the risk or of becoming ill in outbreaks of person-to-person transmission

Variable	Ill	Not ill	Total	OR^*a*^	95% CI	aOR^*b*^	95% CI
Sex							
Male	121	37	158	1.75	1.12–2.75	1.28	0.77–2.13
Female	166	89	255	1		1	
School level							
Infant (>2 and <7 yrs)	171	40	211	1		1	
Primary (≥7 and <13 yrs)	46	6	52	1.79	0.72–4.49	2.16	0.79–5.93
Secondary and Higher (≥13 and <19 yrs)	37	15	52	0.58	0.29–1.15	0.83	0.32–2.19
Adults (≥19 yrs)	33	65	98	0.12	0.07–0.20	0.14	0.08–0.26
Center							
School	213	88	301	1		1	
Summer camp	74	38	112	0.93	0.80–1.09	1.41	0.54–3.64
Season							
Warm months	83	59	142	1		1	
Cold months	204	67	271	2.16	1.40–3.34	2.13	1.23–3.67
Reporting delay							
≤ 2 days	57	37	94	1		1	
>2 days and ≤4 days	39	19	58	1.33	0.67–2.65	1.95	0.68–5.57
>4 days and ≤9 days	107	43	150	1.61	0.94–2.78	3.07	1.21–7.81
>9 days	84	27	111	2.02	1.11–3.68	3.81	1.42–10.23

*^a^*OR, raw odds ratios.

*^b^*aOR, adjusted odds ratios.

In common source outbreaks (Table 3) there was an association between the risk of becoming ill and seasonality, outbreaks in summer camps, being a secondary education or higher student and a delay in reporting of 3 to 4 days. No dose-response was observed between delayed reporting and the risk of becoming ill.

**TABLE 3:** Raw and adjusted odds ratios of the association between the different variables considered and the risk of getting ill in common source outbreaks

Variable	Ill	Not ill	Total	OR^*a*^	95% CI	aOR^*b*^	95% CI
Sex							
Male	67	24	91	2.79	1.57–4.97	2.17	0.94–5.01
Female	66	66	132	1		1	
School level							
Infant (>2 and <7 yrs)	12	30	42	1		1	
Primary (≥7 and <13 yrs)	60	0	60	–^*c*^	–	–	–
Secondary and higher (≥13 and <19 yrs)	26	2	28	32.50	6.65–158.80	44.88	6.70–300.73
Adults (≥19 yrs)	35	58	93	1.51	0.68–3.32	2.11	0.66–6.67
Center							
School	24	59	83	1		1	
Summer camp	109	31	140	2.70	1.9–3.82	9.75	3.35–28.33
Season							
Warm months	81	49	130	1		1	
Cold months	52	41	93	0.77	0.45–1.32	7.42	2.31–23.84
Reporting delay							
≤ 2 days	86	62	148	1		1	
>2 days and ≤4 days	35	25	60	1.01	0.55–1.85	5.38	1.74–16.64
>4 days and ≤9 days	12	3	15	2.88	0.78–10.65	0.40	0.06–2.69
>9 days	0	0					

a OR, raw odds ratios.

b aOR, adjusted odds ratios.

c -, no calculable.

Norovirus GI produced 28.9% of outbreaks (AR 21.57%), 63.2% were due to GII (AR 25.92%) and in 7.9% the etiology was mixed GI/GII (AR 31.22%). Genogroup IV was not detected in any outbreak. The rate ratio (RR) between GI and GII outbreaks was 0.83 (95% CI 0.7–0.94). In outbreaks with person-to-person transmission produced by GI the AR was 15.49% and in those produced by GII the AR was 19.56% (RR 0.79 [95% CI 0.6–0.95]). When transmission was by common source the AR of the outbreaks produced by GI was 35.14% and in those caused by GII was 32.43% (RR 1.08 [95% CI 0. 93–1.26]).

The most frequent genotypes for GI were GI.3 (45.5%), GI.4 (27.3%) and GI.1 (18.2%) and for GII were GII.2 (24%), GII.4 (24%), GII.3 (12%), and GII.6 (12%); in 3 outbreaks (2 of GII and 1 of GI) the genotype was not identified.

## DISCUSSION

The proportion of AGE outbreaks due to norovirus in schools and summer camps found in our study (37.6%) is similar to that found by other authors. In England, 22% of norovirus outbreaks between 2015 and 2020 occurred in educational establishments (18). As this study includes data from 2020, in which activity in schools decreased significantly due to the COVID-19 pandemic, this could explain the differences observed between the studies.

The AR we observed in outbreaks of person-to-person transmission (19.19%) is also similar to that described by other authors. Steele et al. studied norovirus outbreaks reported in the United States. between 2009 and 2017 with person-to-person transmission and found an attack rate of 22% (19) and Matthews et al. in a review of 902 outbreaks reported between 1993 and 2011 found a mean rate of 27% in outbreaks of person-to-person transmission (20).

In our study only one outbreak was waterborne with an attack rate of 64.1% (21), clearly larger that the attack rate in all common source transmission outbreaks (33.42%) or in all studied outbreaks (25.7%). Waterborne outbreaks reported by other authors also show a large number of affected people (22, 23).

We found a higher proportion of women were affected (55.37%). Wikswo et al. in a 2009–2010 United States study found that 71% of persons affected by norovirus outbreaks were women (24). However, other studies did not find women were more affected. Thus, Wang et al. studied norovirus outbreaks in schoolchildren in Shanghai in 2017 and reported that 52.1% of those affected were male (25). Despite the higher percentage of women affected in our study, when we analyzed the risk of becoming ill by sex, we found that the risk was significantly higher in men than in women in all outbreaks. The differences between the sexes could be explained by the fact that unaffected women responded to the survey more frequently than men, although other authors have found that men are less susceptible than women to norovirus (26). When common source outbreaks and person-to-person transmission outbreaks are analyzed separately, this association was not maintained.

The mean number of people affected in each outbreak (34 people per outbreak) is in line with the results found by Lian et al. in a study conducted between 2014 and 2017 in China (mean of 34 people per outbreak) (27) and is not very different from that described by Wikswo et al. in outbreaks of person-to-person transmission in the US in 2009–2010 (mean of 44 people per outbreak) (24) or from the mean of 40 people per outbreak observed by the same authors in 2009–2013 (28). He et al. found a median of 16 persons affected by outbreaks (range 5 to 148) in Shanghai schoolchildren between June 2016 and December 2017 (11).

In our study, the highest proportion of persons affected was observed in early-years education (43.57%), coinciding with the results found by other authors. O'Brien et al. in a study during 2008 and 2009 in the United Kingdom found a higher incidence in children aged < 5 years than in the other age groups (29). Inaida et al. in a study conducted between 2006 and 2009 in Tokyo observed that the group most affected was children aged 0 to 4 years, followed by those aged 5 to 9 years (30), which seems logical, since young children have greater contact with each other, and hand hygiene is worse. However, in studies of outbreaks in schools and summer camps, other studies have found that primary school children were the most affected group. Lian et al. in China between 2014 and 2017 found that 47.98% of affected schoolchildren were in primary school versus 16.86% in early childhood education (27). Wang et al. found that 54.9% of affected children in Shanghai in 2017 were primary school children compared with 39.73% in early childhood education (25) and He et al. in Shanghai between June 2016 and December 2017 found that 61.08% of affected children were in primary school (11).

In common source outbreaks the risk of becoming ill was higher in summer camps than in schools, which may be explained by the fact that in summer camps all meals are made in common and also consume the same water.

In our study, 52.63% of outbreaks occurred in the fall and winter months (October to March), similar to the 68% found by Steele et al. in outbreaks in the US in 2009 (21). Ahmed et al. in a meta-analysis of 293 studies found that 71% of outbreaks occurred in the cold months of the year (October to March in the Northern hemisphere and April to September in the Southern hemisphere) (14). Other studies have also found this seasonal effect (24, 27).

Outbreaks lasted between 1 and 23 days with a mean of 6.11 days (SD 6.08). Lian et al. found a duration of 7.4 days in outbreaks in secondary schools (27). Cheek et al. describe several outbreaks in summer camps lasting 4 to 9 days (31) and Nygård et al. found a duration of 10 days in a waterborne outbreak in a camp in Norway (32).

The delay in reporting was linearly correlated with a longer duration of outbreaks, a result consistent with that observed by He et al. in the study conducted in Shanghai in 2016 and 2017 (11). The fact that the later the outbreak is reported the longer it lasts suggests that rapid action should be taken to control the spread of outbreaks and shorten their duration. Friesema et al. found that rapid adoption of control measures in norovirus outbreaks in nursing homes decreased both the rate of attack and the duration (33).

In our study, the attack rate showed a decreasing exponential relationship with the size of the groups. A similar phenomenon was observed by Tsang et al., who found that in outbreaks that affected households with fewer cohabitants, attack rates were higher than in outbreaks that affected households with more cohabitants and attributed these results to the fact that contact between cohabitants is lower in homes with a greater number of members (26). Brinkhues et al. studied the association between social groups and the prevalence of some infectious diseases, including AGE. They found that large groups with close relationships (such as groups of friends) had a high prevalence of AGE, while in groups in whom the relationship was not so close, the prevalence of AGE was lower (34). Potter et al. observed the same phenomenon when studying the transmission of influenza in schoolchildren, a disease in which contact between people plays an important role, and indicated that groups of schoolchildren are not homogeneous, so it cannot be assumed that contacts are random (35). The lower attack rate found in our study in larger groups can be explained by the segmentation that occurs in large groups, with the risk being heterogeneous in the different subgroups, something that does not occur in small groups whose members have a similar level of exposure.

Norovirus GII was the most frequently identified genogroup. Although the differences were not statistically significant, the risk of being affected by GI was higher than that of being affected by GII in outbreaks with common source transmission, in contrast to what occurred in outbreaks with person-to-person transmission. These results coincide with those found by other authors. Matthews et al. in a review of norovirus outbreaks between 1983 and 2010 found that 76% of outbreaks in day care centers and schools were caused by GII and that GI was more frequently associated with outbreaks with common source transmission than with those with person-to-person transmission (20).

Our study has limitations. First, possible selection bias among the individuals completing the epidemiological survey, since the number of unaffected respondents is low compared to the number of affected persons (216 and 420, respectively). Second, by conducting separate analyses of outbreaks of person-to-person transmission and common source outbreaks, the number of persons included in the analysis was reduced, with the consequent loss of statistical power.

A strength of the study was that it was an analysis in real conditions of epidemiological surveillance covering all Catalonia over a period of several years.

### Conclusions.

School level, the type of center and delayed reporting showed distinct associations with the risk of becoming ill in outbreaks of person-to-person transmission and common source outbreaks, suggesting the desirability of analyzing norovirus AGE outbreaks depending on the type of transmission. In outbreaks with a large number of exposed persons, the possible existence of subgroups with nonhomogenous exposure levels should be considered, because the overall attack rate does not reflect the attack rate of the different subgroups. A noteworthy aspect of this study carried out in schools and summer camps is that the delay in reporting was associated with a longer duration of outbreaks, which reinforces the importance of early reporting in this type of centers.

## MATERIALS AND METHODS

Prospective study of outbreaks reported in Catalonia from January 2017 to December 2019 in schools and summer camps. The clustering of ≥ 2 cases of AGE in schools or summer camps in which norovirus was identified in clinical samples by real-time semiquantitative reverse transcription PCR (RTqPCR) was considered an outbreak.

Feces were collected to identify norovirus genogroups I, II and IV by RTqPCR. Samples were analyzed at the Microbiology Laboratory, Vall d'Hebron Hospital and Public Health Agency of Barcelona laboratory. The specific primers described by Kageyama et al. were used to detect norovirus GI and GII (36). A modification of the primer described by Farkas et al. (37) and Kageyama et al. (36) was used to detect norovirus GIV.

For each outbreak, the number of exposed and affected people, the type of transmission (person-to-person or by common source), the date of onset and the end of the outbreak and the date when the Epidemiological Surveillance unit was notified were collected. Percentages were compared using a linear trend chi-square test. Attack rates (AR) were calculated considering the total number of affected and exposed persons in the outbreaks and according to the causal genogroup; the rate ratio (RR) and 95% confidence intervals (CI) globally and according to type of transmission were calculated.

To study the associations between sex, age, type of center, delay in reporting and season and the risk of becoming ill, crude odds ratios (OR) and adjusted odds ratios (aOR) were estimated with their 95% CI. To estimate the aOR, multivariate analysis was performed by logistic regression, adjusting using the backward stepwise procedure with a cutoff point of <0.2.

The correlation between the delay in reporting and the duration of the outbreak was estimated using Pearson's correlation coefficient and 95% CI. For studied the relation between the size of the affected group and the attack rate was used a model of logarithmic transformation both the dependent and independent variables (log-log model) and the correlation between the transformed variables was estimated using Pearson’s correlation coefficient and 95% IC.

The study was conducted according to the guidelines of the Declaration of Helsinki, regulations of the Public Health Agency of Catalonia and ethical protocols established.

The study was approved by the University of Barcelona Bioethics Commission (Institutional Review Board IRB00003099) on April 12, 2016.

We declare that the Bioethics Committee of University of Barcelona approved the waiver for informed consent.

All data used in the analysis were collected during routine public health surveillance activities as part of the legislated mandate of the Health Department of Catalonia, which is officially authorized to receive, treat and temporarily store personal data in the case of infectious disease. All data were fully anonymized. All study activities formed part of the public health surveillance tasks. Law regulates these activities and informed consent should not be necessary.

### Data availability.

The data sets generated during the current study are available in the Mendeley Data repository, https://data.mendeley.com/datasets/3ktswshxgv/1.
